# Estimation of the LDL subclasses in ischemic stroke as a risk factor in a Chinese population

**DOI:** 10.1186/s12883-020-01989-6

**Published:** 2020-11-13

**Authors:** Ruisheng Duan, Wenjun Xue, Kunpeng Wang, Nan Yin, Hongyu Hao, Hongshan Chu, Lijun Wang, Peng Meng, Le Diao

**Affiliations:** 1 Department of Neurology, Hebei General Hospital, Shijiazhuang, 050000 Hebei China; 2 Department of Neurology, the First People’s Hospital of Pingdingshan, Henan 467000 Pingdingshan, China; 3 Department of Neurosurgery, the Affiliated Hospital of Chengde Medical University, Chengde, 067000 Hebei China; 4 Department of Medicine, Shanghai Zhangjiang institute of Medical innovation, Biotecan Pharmaceuticals co., ltd., Shanghai, 201204 China

**Keywords:** LDL subclasses, Small dense LDL, Ischemic stroke, Cerebrovascular disease

## Abstract

**Background:**

Acute ischemic stroke (AIS) is one of the leading causes of mortality and long-term disability worldwide. Our study aims to clarify the role of low-density lipoproteins (LDL) subclasses in the occurrence of AIS and develop a risk xprediction model based on these characteristics to identify high-risk people.

**Methods:**

Five hundred and sixty-six patients with AIS and 197 non-AIS controls were included in this study. Serum lipids and other baseline characteristics including fasting blood glucose (GLU), serum creatinine (Scr), and blood pressure were investigated in relation to occurrence of AIS. The LDL subfractions were classified and measured with the Lipoprint System by a polyacrylamide gel electrophoresis technique.

**Results:**

Levels of LDL-3, LDL-4 and LDL-5 subclasses were significantly higher in the AIS group compared to the non-AIS group and lower level of LDL-1 was prevalent in the AIS patients. Consistently, Spearman correlation coefficient demonstrated that sd-demonevels, especially LDL-3 and LDL-4 levels, were significantly positively correlated with AIS. Furthermore, there is a significant positive correlation between small dense LDL (sd-LDL, that is LDL-3 to 7) levels and serum lipids including total cholesterol (TC), Low density lipoprotein cholesterol (LDL-C), and Triglyceride (TG). Increased LDL-3 and LDL-4 as well as decreased LDL-1 and LDL-2 were correlated to the occurrence of AIS, even in the people with normal LDL-C levels. A new prediction model including 12 variables can accurately predict the AIS risk in Chinese patients (AUC = 0.82 ± 0.04).

**Conclusions:**

Levels of LDL subclasses should be considered in addition to serum LDL-C in assessment and management of AIS. A new prediction model based on clinical variables including LDL subtractions can help clinicians identify high of AIS, even in the people with norm.

## Background

Stroke is known as an acute cerebrovascular disorder with a sudden loss or deterioration of brain function, resulting from ischemic infarction or intracranial hemorrhage. Acute ischemic stroke (AIS) is one of the leading causes of mortality and long-term disability worldwide, which accounts for approximately 80% of all strokes. According to the Global Burden of Disease (GBD) Study 2016 estimates of stroke Incidence, China had the greatest estimated lifetime risk and bore the biggest stroke burden in the world. Meanwhile, the stroke burden in China has been increasing over the past 30 years, and remains specifically high in rural areas, with the bulk of this burden occurring in the northern and central regions [1].

As a multifactorial disease, stroke occurs due to various genetic and environmental factors like hypertension, smoking, and alcohol use [2, 3]. One such vital risk factor responsible for the development of stroke is atherosclerosis (AS) of the cerebral circulation, which is closely associated with the abnormalities of serum lipids and lipoproteins, including elevated triglyceride levels, reduced high-density lipoproteins (HDL) cholesterol concentrations and increased low-density lipoproteins (LDL) cholesterol concentrations [4]. Specifically, LDL comprises multiple distinct subclasses, defined as LDL-1 to LDL-7 in gradient gel electrophoresis, which differ in size, density, physicochemical composition, metabolic behavior and atherogenicity. Among them, LDL-3 to 7 is also named as small dense LDL (sd-LDL) according to their size and density, whereas LDL-1 and 2 are defined as large LDL correspondingly [4, 5].

According to the National Cholesterol Education Program Adult Treatment Panel III (NCEP III), high level of sd-LDL is an established risk factor for cardiovascular disease (CVD) [6], however, their clinical significance for AIS is underappreciated compared to CVD. Besides, considering the complex etiology of stroke, studies that investigate the association of various risk factors and AIS should be conducted in populations with different genetic and environmental backgrounds. Here, we evaluated serum lipid profile and LDL subtractions in AIS patients and non-AIS controls, analyzed the correlation of clinical characteristics and AIS risk in a Chinese population from northern and central regions, and established a predictive model for AIS risk with selected variances.

## Methods

### Study population

The study group consisted of 566 hospitalized patients (318 males and 248 females; mean age, 66.1 ± 13.1 years) with the first onset of AIS. The patients were recruited from department of neurology in Hebei General Hospital and the First People’s Hospital of Pingdingshan and department of Neurosurgery in the Affiliated Hospital of Chengde Medical University, between December 2016 and March 2018. The diagnosis of ischemic stroke was based on current guidelines, and computed tomography (CT) or magnetic resonance imaging (MRI) were performed by experienced neurologists to confirm the diagnosis of ischemic stroke and to rule out hemorrhagic stroke.

On admission, essential characteristics and risk factors of patients were collected and evaluated, including age, sex, height, weight, smoking, alcohol use, blood pressure and blood glucose (GLU). Patients included in this study did not use any lipid-lowering therapy. Co-morbidities were also evaluated as follows: hypertension (HT), according to a previous diagnosis, or measured systolic blood pressure (SBP) ≥140 mmHg, and/or diastolic blood pressure (DBP) ≥90 mmHg; Presence of cardiovascular disease (previous myocardial infarction, coronary artery disease, valvular heart disease, cardiomyopathy or arrhythmia) and diabetes mellitus (DM), according to a previous diagnosis or current medication for diabetes were also recorded.

Corresponding controls (*n* = 197; 91 males and 106 females; mean age, 64.7 ± 13.7 years) were selected based on the absence of neurological diseases as well as the same exclusion criteria as described for the cases in clinic patients with non-stroke neurological disorders.

Every individual was informed about the aims of the study and provided written consent before participation. Written informed consent was obtained from a relative in the case of patients with impaired cognition.

### Blood collection and analysis

Blood samples were collected in EDTA tubes following overnight fasting for at least 12 h, and analyzed by a solid-phase, enzyme-labeled chemiluminescent immunometric assay on Siemens Advia 2400 with the manufacturer’s reagents as directed to detect blood GLU, serum creatinine (Scr), Triglyceride (TG), total cholesterol (TC), High density lipoprotein cholesterol (HDL-C), and Low density lipoprotein cholesterol (LDL-C).

### Low-density lipoprotein analysis

Blood samples were also used for LDL subfraction analysis. LDL subgroups were classified and measured with the Lipoprint System (Quantimetrix Corporation, Redondo Beach, CA, USA) according to the manufacturer’s instructions as previously described [7, 8]. The clinical application of this system is approved by the Food and Drug Administration (FDA). This system separates LDL subfractions by a polyacrylamide gel electrophoresis technique and gels were scanned to determine the relative area of each lipoprotein subfraction after electrophoresis. Based on net surface charge and size, various stained LDL subfractions are classified according to their mobilities in the gel. By this analysis, LDL was divided into 7 subfractions (LDL-1 to LDL-7).

### Statistical analysis

Statistical analyses were performed using Python software (Version 3.6). Parametric statistics (t-test) were used for normally distributed data. Nonparametric Mann–Whitney U test were used for non-normally distributed data and expressed as median (25, 75% IQR). Categorical variables were presented by numbers or proportions, and difference in distribution between groups were analyzed by Chi-square or the Fisher’s exact test, as appropriate. If the data are not normally distributed, correlation analysis was performed using the Spearman correlation coefficient. The eXtreme gradient boosting (XGBoost) Classifier decision trees model (Xgboost 0.81), Random Forest Classifier, Logistic Regression, AdaBoost Classifier were performed to estimate the stroke risk with variables. The k-fold cross-validation (k = 5) was implemented to determine the performance. Related data were presented as receiver operating characteristics (ROC) curves and area under the curve (AUC). An AUC value > 0.5 indicated better predictive values; the closer the AUC to 1, the better the model performance.

## Results

### Clinical and laboratory characteristics of study participants

A total of 566 patients were included in the AIS group and 197 age-matched non-AIS patients were set as control group. The baseline characteristics of both study groups are shown in Table 1. The BMI, the proportion of patients with hypertension, diabetes mellitus, metabolic syndrome, and cardiovascular disease, SBP, DBP, Scr, LDL-C, TG, Apolipoprotein A (ApoA), and sd-LDL were significantly higher than those in the control group (*P* <  0.05). Smoking, and alcohol consumption, and GLU, TC, and Apolipoprotein B (ApoB) did not differ between the two groups, while the HDL-C concentration was significantly higher in the control group.

**Table 1:** Clinical and laboratory characteristics in all subjects

Characteristics	AIS (*n* = 566)	Controls (*n* = 197)	*P* value *
Age (years)	66 (57, 77)	67 (51, 76)	0.114
Sex (M/F)	318/248	91/106	0.016 *
BMI (kg/m^2^)	24.4 (22.4, 27.4)	23.4 (21.4, 25.3)	< 0.001 *
Smoking (%)	75 (13.3)	19 (9.6)	0.209
Alcohol use (%)	78 (13.8)	18 (9.1)	0.105
H-type hypertension (%)	103 (18.2)	18 (9.1)	0.002 *
Hypertension (%)	430 (76.0)	79 (40.1)	< 0.001 *
Diabetes mellitus (%)	155 (27.4)	17 (8.6)	< 0.001 *
Metabolic syndrome (%)	115 (20.3)	27 (13.7)	0.043 *
Cardiovascular disease (%)	464 (82.0)	83 (42.1)	< 0.001 *
SBP (mmHg)	139.5 (127, 156)	123 (121, 140)	< 0.001 *
DBP (mmHg)	84 (77, 94)	78 (70, 87)	< 0.001 *
GLU (mmol/L)	5.3 (4.7, 6.2)	5.2 (4.7, 6.1)	0.343
SCr (μmol/L)	69.9 (59, 83)	63 (53, 74)	< 0.001 *
TC (mmol/L)	4.2 (3.5, 5.1)	4.2 (3.5, 4.9)	0.228
LDL-C (mmol/L)	2.5 (1.9, 3.1)	2.3 (1.8, 2.8)	0.003 *
HDL-C (mmol/L)	1.1 (0.8, 1.3)	1.2 (0.9, 1.6)	< 0.001 *
TG (mmol/L)	1.4 (1.0, 2.1)	1.2 (0.9, 1.5)	< 0.001 *
ApoA (mmol/L)	1.2 (1.1, 1.4)	0.9 (0.8, 1.2)	< 0.001 *
ApoB (mmol/L)	0.8 (0.6, 0.9)	0.8 (0.6, 0.9)	0.494
sd-LDL (mg/dL)	7 (2, 17)	3 (1, 6)	< 0.001 *

*AIS* Acute ischemic stroke, *M* Male, *F* Female, *BMI* Body mass index, *SBP* Systolic blood pressure, *DBP* Diastolic blood pressure, *GLU* Fasting blood glucose, *Scr* Serum creatinine, *TC* Total cholesterol, *LDL-C* Low density lipoprotein cholesterol, *HDL-C* High density lipoprotein cholesterol, *TG* Triglyceride, *ApoA* Apolipoprotein A, *ApoB* Apolipoprotein B, *sd-LDL* Small dense LDL; *p <  0.05

### Distribution of LDL subtractions in AIS patients and controls

As shown in Table 2, levels of LDL-3, LDL-4 and LDL-5 subclasses were significantly higher in the AIS group compared to the control group (*p* <  0.05). In contrast, LDL-1 was significantly lower in the AIS group, while there was no difference between the AIS and control groups in terms of LDL-2, LDL-6 and LDL-7 (*p* > 0.05).

**Table 2:** The distribution of LDL subclasses in AIS patients and controls

LDL subclasses	AIS (*n* = 566)	Controls (*n* = 197)	*P* value *
LDL-1 (mg/dL)	21.5 (15, 29)	23 (17, 30.5)	0.021 *
LDL-2 (mg/dL)	18 (13, 25)	18 (13, 24)	0.318
LDL-3 (mg/dL)	7 (2, 13)	3 (1, 6)	< 0.001 *
LDL-4 (mg/dL)	0 (0, 3)	0 (0, 0)	< 0.001 *
LDL-5 (mg/dL)	0 (0, 0)	0 (0, 0)	0.032 *
LDL-6 (mg/dL)	0 (0, 0)	0 (0, 0)	0.117
LDL-7 (mg/dL)	0 (0, 0)	0 (0, 0)	0.206

*LDL* Low density lipoprotein; *p <  0.05

### Correlations of clinical characteristics and AIS risk

**Fig. 1:**
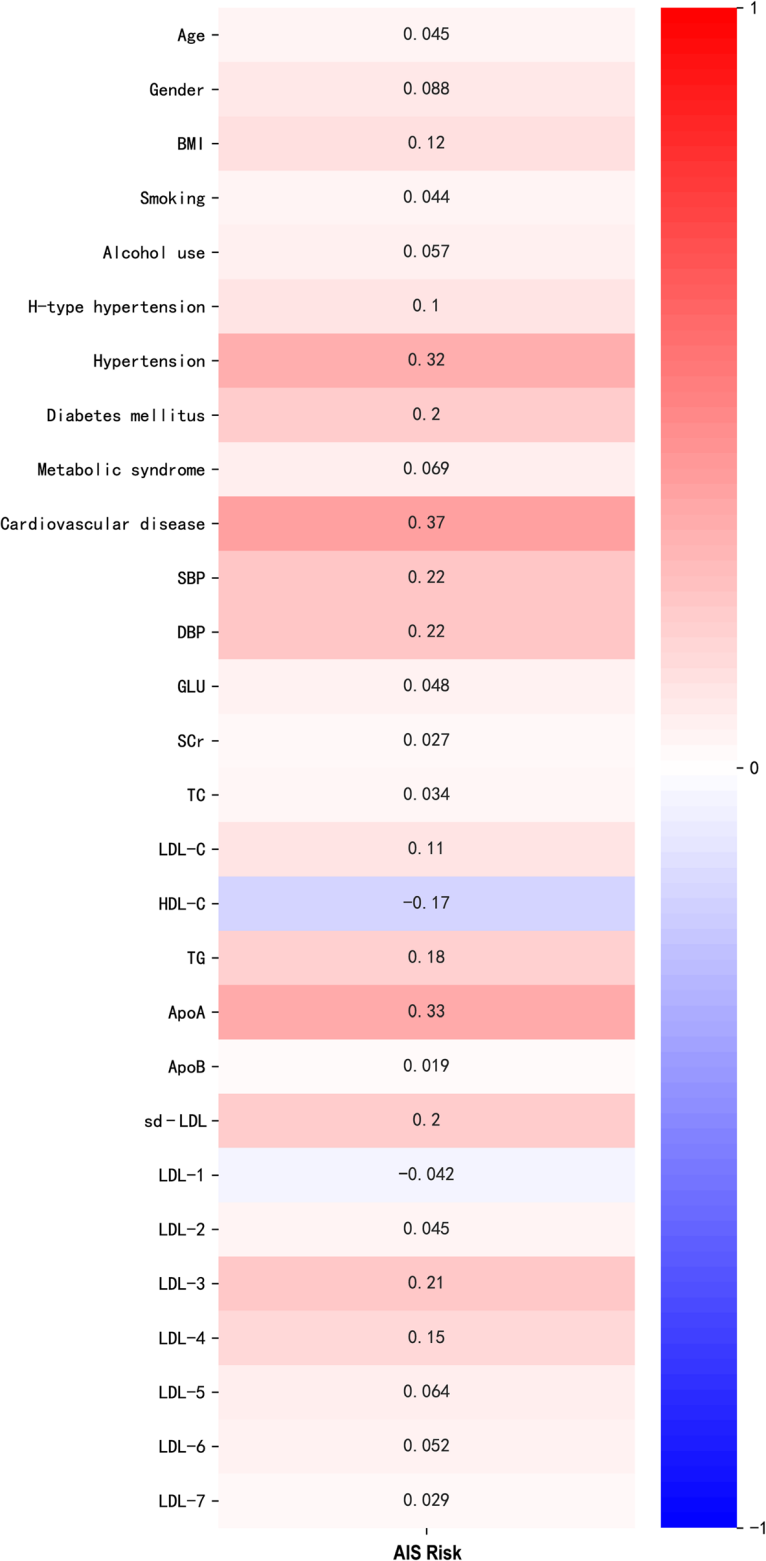
Heatmap showing the correlation between clinical characteristics and AIS risk

Spearman correlation coefficient was used to assess the correlation between clinical characteristics and AIS risk, and heatmap was drawn accordingly (Fig. 1). It was found that HDL-C was negatively correlated with presence of AIS in the study cohorts (*r* = − 0.17, *P* <  0.001). A significant positive correlation was found between AIS and several clinical and laboratory features including BMI, hypertension, diabetes mellitus, cardiovascular disease, SBP, DBP, LDL-C, TG, ApoA, sd-LDL, LDL-3 and LDL-4 (r > 0.1, *P* <  0.001).

### Correlations of LDL subtractions and LDL-C

We next performed Spearman correlation coefficient to investigate the correlations between LDL-C and its subclasses (Table 3). As a result, there was a significant positive correlation between LDL-C and LDL-1 (*r* = 0.540, *p* <  0.05), LDL-2 (*r* = 0.547, *p* <  0.05), LDL-3 (*r* = 0.343, *p* <  0.05) and LDL-4 (*r* = 0.227, *p* <  0.05). With regard to the correlation among LDL subtypes, LDL-1 was found positively correlated with LDL-2 (*r* = 0.485, *p* <  0.05), but negatively correlated with the rest LDL subtypes, whereas LDL-2 was positively correlated with LDL-3 (*r* = 0.611, *p* <  0.05) and LDL-4 (*r* = 0.230, *p* <  0.05) besides LDL-1, but negatively correlated with LDL-5 to 7. For the LDL-3 to 7, so called sd-LDL, there is a positive correlation between each other in most cases except for LDL-3 and LDL-7 (*r* = − 0.019, *p* = 0.599).

**Table 3:** Correlation analysis of LDL-C and its subclasses by Spearman correlation method

	LDL-C	LDL-1	LDL-2	LDL-3	LDL-4	LDL-5	LDL-6	LDL-7
LDL-1	0.540*		0.485*	− 0.111*	− 0.279*	− 0.260*	−0.181*	− 0.056
LDL-2	0.547*			0.611*	0.230*	−0.066	−0.155*	− 0.077*
LDL-3	0.343*				0.722*	0.325*	0.089*	−0.019
LDL-4	0.227*					0.543*	0.247*	0.057
LDL-5	0.063						0.495*	0.163*
LDL-6	−0.046							0.345*
LDL-7	−0.038							

*p <  0.05

### Correlation analysis between sd-LDL and serum lipids

**Fig. 2:**
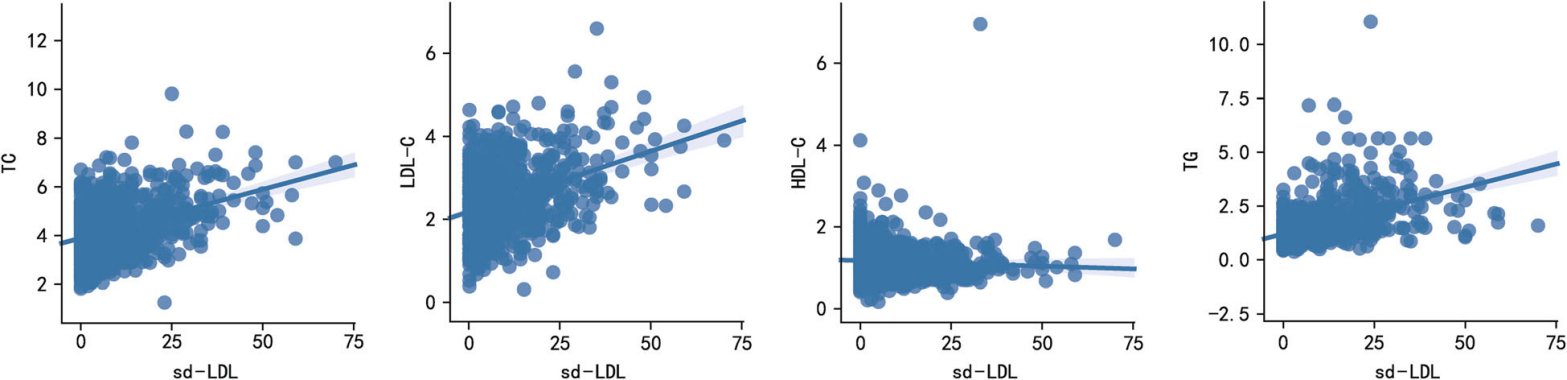
Correlation between sd-LDL and serum lipids including TC, LDL-C, and TG

Spearman correlation coefficient showed a significant positive correlation between sd-LDL levels and serum lipids including TC, LDL-C, and TG, as shown in Table 4 and Fig. 2. Sd-LDL was negatively correlated with HDL-C in control group and all subjects, but not in AIS group.

**Table 4:** Correlation coefficient between sdLDL-C and serum lipids by Spearman correlation method

Serum lipids	All subjects (*n* = 763)	AIS (*n* = 566)	Controls (*n* = 197)
TC	0.315 *	0.402 *	0.037
LDL-C	0.310 *	0.345 *	0.159 *
HDL-C	−0.138 *	0.067	−0.216 *
TG	0.536 *	0.593 *	0.221 *

*p <  0.05

### Clinical and laboratory characteristics of study participants with normal LDL-C levels

In AIS group, the sd-LDL level was significantly higher in the high LDL-C group of patients than in the normal LDL-C group (*P* <  0.001) (Table 5). Additionally, although there was no significant difference in GLU and LDL-C levels between the AIS group with normal LDL-C levels and the control group (*P* > 0.05), the other laboratory characteristics, including blood pressure, Scr, serum lipids, and LDL subclasses, differed significantly between the two groups, as shown in Table 6.

**Table 5:** Comparison of sd-LDL between the normal LDL-C group (LDL-C < 2.59 mmol/ L) and high LDL-C (LDL-C ≥ 2.59 mmol/ L) group of patients with AIS

	Normal (*n* = 314)	High (*n* = 252)	*P* value *
sdLDL (mg/dL)	5 (2, 11.2)	11.5 (4.8, 23)	< 0.001 *

**Table 6:** Comparison of laboratory characteristics between the AIS group and control group with normal LDL-C levels (< 2.59 mmol/ L)

Characteristics	AIS (*n* = 314)	Controls (*n* = 127)	*P* value *
SBP (mmHg)	137.5 (127.3, 154)	123 (123, 135.5)	< 0.001 *
DBP (mmHg)	84 (77, 93)	78 (67.5, 86)	< 0.001 *
GLU (mmol/L)	5.2 (4.6, 6.2)	5.2 (4.6, 6.4)	0.356
SCr (μmol/L)	68.7 (56, 83.4)	63 (53, 74.5)	0.028 *
TC (mmol/L)	3.5 (3.1, 4)	3.7 (3.3, 4.2)	0.016 *
LDL-C (mmol/L)	1.9 (1.6, 2.3)	1.9 (1.6, 2.3)	0.337
HDL-C (mmol/L)	0.9 (0.7, 1.2)	1.1 (0.9, 1.6)	< 0.001 *
TG (mmol/L)	1.3 (1, 1.8)	1.1 (0.9, 1.4)	< 0.001 *
ApoA (mmol/L)	1.2 (1.1, 1.3)	0.8 (0.7, 1)	< 0.001 *
ApoB (mmol/L)	0.6 (0.5, 0.8)	0.7 (0.6, 0.7)	< 0.001 *
sdLDL (mg/dL)	5 (2, 11.2)	3 (1, 5.5)	< 0.001 *
LDL-1 (mg/dL)	17 (13, 23)	22 (15, 28)	< 0.001 *
LDL-2 (mg/dL)	15 (10, 20)	16 (11, 21)	0.043 *
LDL-3 (mg/dL)	5 (2, 9)	3 (1, 5)	< 0.001 *
LDL-4 (mg/dL)	0 (0, 2)	0 (0, 0)	< 0.001 *
LDL-5 (mg/dL)	0 (0, 0)	0 (0, 0)	0.065
LDL-6 (mg/dL)	0 (0, 0)	0 (0, 0)	0.170
LDL-7 (mg/dL)	0 (0, 0)	0 (0, 0)	0.185

*p < 0.05

### Prediction of AIS risk by laboratory characteristics with an established model

**Fig. 3:**
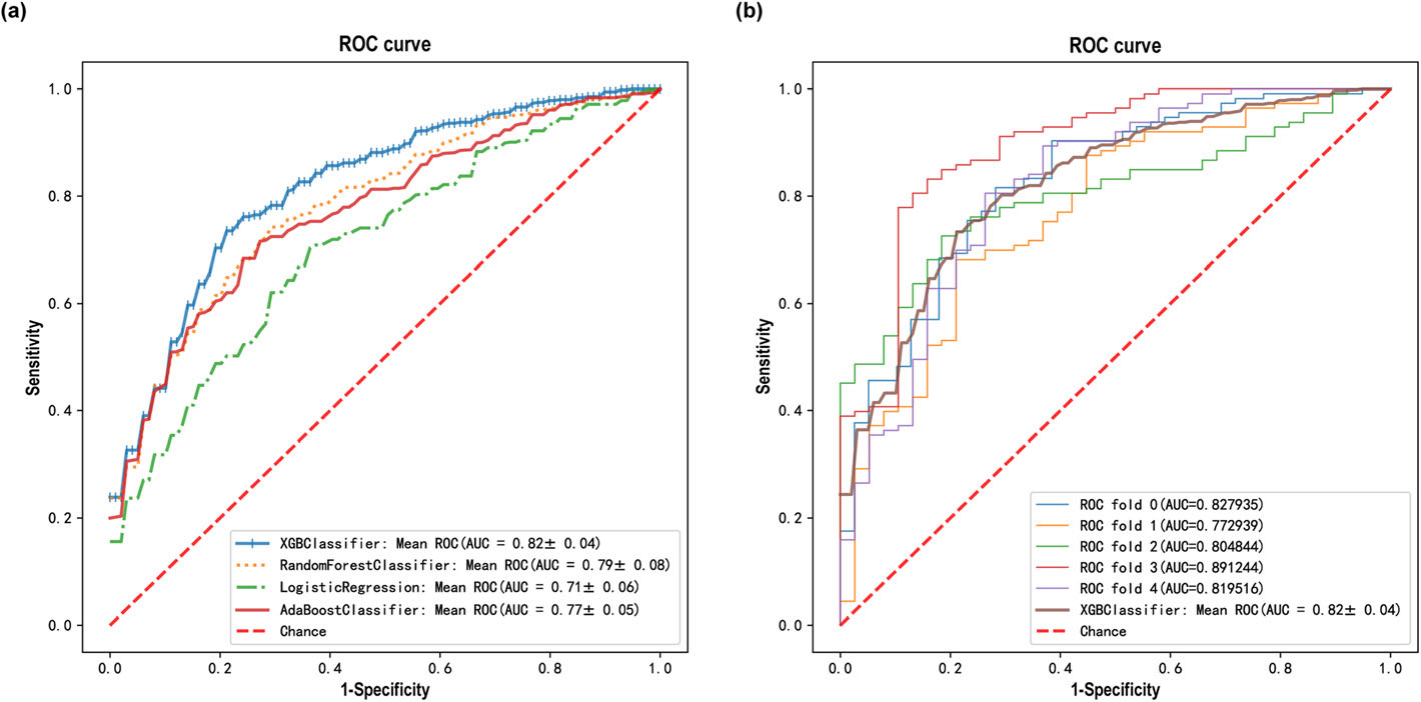
Prediction of the relationship between laboratory characteristics and AIS with an established model. **a** Receiver operating characteristic (ROC) curve and area under the curve (AUC) for multiple classifiers including XGBoost, Random Forest, Logistic Regression, and AdaBoost. **b** The k-fold cross-validated (k = 5) ROC curves for XGBoost classifier

Considering the correlations of laboratory characteristics with AIS, a risk prediction model was established accordingly to identify people most likely to develop AIS (Fig. 3). Several typical and recent artificial intelligence (AI) algorithms were assessed in the context of AIS, and the XGBoost stood out as our base model due to its high performance (AUC = 0.82 ± 0.04) (Fig. 3a). As a frequently used resampling method, k-fold cross-validation was employed to estimate the performance of the XGBoost classifier (Fig. 3b). The new prediction model contained 12 variables (ie, Scr, SBP, DBP, TG, TC, LDL-C, HDL-C, LDL-1, LDL-2, LDL-3, LDL-4, LDL-5) with a cut-off value of 0.5.

## Discussions

In China, stroke was still the top leading cause of years of life lost (YLLs) and disability-adjusted life-years (DALYs) in 2017 [9]. Various risk factors have been implicated with stoke, and one of the most important traditional risk factors is the elevation of LDL-C, consisting of 7 subtractions, defined as LDL-1 to LDL-7. Existing data on the relationship between LDL subclasses and AIS are however equivocal. The objective of this study was to examine the contribution of LDL subtractions to AIS as a risk factor in a Chinese population and to develop a risk prediction model based on these characteristics to identify people most likely to develop AIS.

The mechanisms related to the atherogenicity of sd-LDL have long been recognized, such as penetrating the arterial wall more easily, being more susceptible to be oxidized or binding to the glycosaminoglycans in the arterial wall more readily, and so on, thus it has been stated in several studies as an independent and more potent risk factor for coronary and peripheral artery disease as well as carotid atherosclerosis, compared to LDL-C [10–12]. More recent study in a Japanese population of 345 men ≥65 years old with stable coronary artery disease showed that small dense Low density lipoprotein cholesterol (sdLDL-C) is a more effective secondary biomarker for cardiovascular events than LDL-C [13]. Another study compared the values of six LDL-related variables for predicting the severity of coronary artery disease (CAD), using untreated Chinese patients undergoing coronary angiography (CAG). The predicting value of CAD turned out to be sd-LDL > oxidized-low density lipoprotein (ox-LDL) > ApoB > non-HDL-C > LDL-C > lipoprotein (a) and all variables existed as independent risk factors for the severity of CAD in multivariate logistic analysis [14]. Chaudhary et al. showed that elevated LDL-4 level is an independent predictor of severe CAD (AUC = 0.62, *p* <  0.0001) with a cut-off value of > 16.9 mg/dL, a sensitivity of 53%, and specificity of 79%, respectively [15].

To date, the studies on the relationship between LDL subtractions and AIS are still limited. In this study, we found a significant relationship between sd-LDL and AIS. Sd-LDL was significantly higher in the AIS group than those in the control group and Spearman correlation coefficient showed that sd-LDL levels, particularly LDL-3 and LDL-4 levels, were significantly positively correlated with AIS. Besides, LDL-3, LDL-4 and LDL-5 levels were significantly higher in the AIS group compared to the control group, while LDL-1 was significantly lower. In terms of certain LDL particles, our findings are different from the other two studies by Zeljkovic et al. and Kayran et al. The former Serbia group examined LDL subclasses in 200 AIS patients as well as 162 controls, showing that AIS patients had ignificantly more LDL III and IVb subclasses (sd-LDL), but less large LDL I and II particles [16]. The latter, however, recruited 110 AIS patients and 60 healthy controls from Turkey in the study, and reported that apart from LDL-3 and LDL-4, the level of large LDL-2 particles was also significantly higher in AIS patients [17]. The distribution of large LDLs (LDL-1 and LDL-2) between AIS and control group is in controversy though, all three studies demonstrated a significant elevation of sd-LDLs, especially LDL-3 and LDL-4 levels in the AIS patients.

Additionally, the correlation of LDL subtractions among each other and the relationship of sd-LDL and serum lipids were assessed in our study. Consistent with the lower distribution of LDL-1 in AIS group, LDL-1 was found negatively correlated with the sd-LDL particles, LDL-3 to 7, suggesting lower potential atherogenicity of this particle. By contrast, LDL-2 was positively correlated with LDL-3 and LDL-4, the main atherogenic particles of sd-LDL, which explains the slightly higher distribution of LDL-2 in AIS group and is consistent with the findings by Kayran et al. Considering the correlation coefficients of LDL-1 and LDL-2 with LDL-C, it is highly recommended that not only LDL-C level but also the LDL subtractions should be taken into consideration for the management of AIS. Although our data suggest the lower atherogenicity of LDL-1, other in vivo studies indicate that LDL-1 is more atherogenic than LDL-2, due to its longer half-life, which renders the particle more susceptible to uptake by the scavenger LDL receptor on macrophages [18]. In this case, further studies with larger sample sizes are needed to clarify the function of each LDL subtraction, especially LDL-1 and LDL-2, in AIS development.

Sd-LDL is often accompanied by increased TG, ApoB and decreased HDL levels [10]. Multiple linear regressions also showed that in CAD and metabolic syndrome patients, TG and TC were the most important determinants of serum sd-LDL concentrations [19]. Consequently, correlation coefficient between sd-LDL and serum lipids were analyzed. As a result, there is a significant positive correlation between sd-LDL levels and serum lipids including TC, LDL-C, and TG, which is consistent with findings reported by other groups.

LDL-C elevation has long been identified as a risk factor for AIS, however, AIS still occurs in people with normal LDL-C, suggesting it is not a sufficient biomarker for AIS prediction. Indeed, our data illustrated that sd-LDL levels in the high LDL-C group of AIS patients was higher than that in the normal group (14.81 ± 13.15 vs. 8.03 ± 8.71, *P* <  0.001). Moreover, the levels of sd-LDL differed significantly between the AIS group and the control group when the level of LDL-C is normal (*P* <  0.05). Intriguingly, large LDL particles (LDL-1 and LDL-2) were significantly lower while LDL-3 and LDL-4, the main atherogenic particles of sd-LDL, were significantly higher in the AIS group. In addition, the other laboratory characteristics, including blood pressure, Scr, TC, HDL-C, TG, ApoA and ApoB also differed significantly between the two groups, indicating that the LDL-C levels alone does not accurately reflect risk to develop AIS. Studies in 22 countries have shown that hypertension is the main risk factor for stroke patients [20]. A high Scr concentration within the normal range is a marker for increased risk of cerebrovascular disease in both normotensive and hypertensive subjects [21]. Taken together, these observations may suggest that in patients with normal LDL-C levels, we need to consider other factors such as blood pressure, Scr, and LDL subtractions to determine the risk of AIS.

Since even in those with normal LDL-C levels, the risk of AIS still exists and impacts of other features cannot be ruled out, a prediction model including most of the risk factors should be established for better prevention of the disease and reducing the associated burden. On the basis of previous studies and the data of this study, considering that it can be widely used in patients, especially in patients with normal LDL-C but still with high risk of AIS, we established a new prediction model including 12 stroke risk factors, namely Scr, SBP, DBP, TG, TC, LDL-C, HDL-C, LDL-1, LDL-2, LDL-3, LDL-4 and LDL-5. The prediction abilities of the four models of XGBoost, Random Forest, Logistic Regression, and AdaBoost classifier were assessed in this study. All models had AUC values above 0.70, and XGBoost achieved the best performance, exhibiting an average AUC of 0.82. It is generally considered that if the AUC of the model is greater than 0.70, the model has high accuracy [22]. The XGboost algorithm is a widely used machine learning method that can build complex models and make accurate decisions when given adequate data [23]. The accuracy and high efficiency of XGBoost make it show excellent performance in clinical research, especially in the field of vascular diseases [23–25]. The new prediction model can accurately predict the AIS risk in Chinese patients and all variables involved in the model can be simple and easily obtained on admission. The cut-off point was defined as 0.5, and an output value > 0.5 indicated AIS risk, which is easy for clinicians to accept and determine the most appropriate medical treatment for prevention.

## Conclusions

In summary, our work analyzed the correlation of LDL subtractions with AIS. The results showed that LDL-3 and LDL-4 are positively correlated with AIS while LDL-1 and LDL-2 are negatively correlated with the disease even in the people with normal LDL-C levels. This finding indicates that levels of LDL subclasses should be considered in addition to serum LDL-C in assessment and management of AIS. Most importantly, a new prediction model including 12 variables were generated, which can help clinicians identify high-risk patients so that proper prevention measures can be taken to ease the potential burden and reduce suffering.

## Data Availability

All data generated or analyzed during this study are included in this published article.

## Abbreviations

AIS: Acute ischemic stroke
LDL: Low-density lipoproteins
GLU: Glucose
Scr: Serum creatinine
sd-LDL: Small dense LDL
TC: Total cholesterol
LDL-C: Low density lipoprotein cholesterol
TG: Triglyceride
GBD: Global Burden of Disease
AS: Atherosclerosis
HDL: High-density lipoproteins
NCEP III: National Cholesterol Education Program Adult Treatment Panel III
CVD: Cardiovascular disease
CT: Computed tomography
MRI: Magnetic resonance imaging
HT: Hypertension
SBP: Systolic blood pressure
DBP: Diastolic blood pressure
DM: Diabetes mellitus
HDL-C: High density lipoprotein cholesterol
FDA: Food and Drug Administration
ROC: Receiver operating characteristics
AUC: Area under the curve
ApoA: Apolipoprotein A
ApoB: Apolipoprotein B
AI: Artificial intelligence
XGBoost: eXtreme gradient boosting
YLLs: Years of life lost
DALYs: Disability-adjusted life-years
sdLDL-C: Small dense Low density lipoprotein cholesterol
CAG: Coronary angiography
ox-LDL: Oxidized-low density lipoprotein
